# Comparison of radiological abnormalities between the jugular bulb and the vestibular aqueduct in patients with Ménière’s disease

**DOI:** 10.3389/fneur.2023.1184232

**Published:** 2023-05-12

**Authors:** Kaijun Xia, Ping Lei, Yingzhao Liu, Jing Li, Miao Wang, Yangming Leng, Bo Liu

**Affiliations:** ^1^Department of Otolaryngology Head and Neck Surgery, Union Hospital, Tongji Medical College, Huazhong University of Science and Technology, Wuhan, China; ^2^Department of Radiology, Union Hospital, Tongji Medical College, Huazhong University of Science and Technology, Wuhan, China

**Keywords:** Meniere’s disease, vestibular aqueduct (VA), jugular bulb, computed tomography, endolymphatic hydrops

## Abstract

**Objective:**

Anatomical variations of the inner ear may contribute to the development of Ménière’s disease (MD), which is a complex inner ear disorder histopathologically characterized by idiopathic endolymphatic hydrops (ELH). Abnormalities of the vestibular aqueduct (VA) and the jugular bulb (JB) have been suggested as predisposing factors. Yet, few studies have investigated the correlation between JB abnormalities and VA variations as well as its clinical relevance in these patients. In this retrospective study, we investigated the differences in the incidence of radiological abnormalities of the VA and JB in patients with definite MD.

**Methods:**

Anatomical variations of JB and VA were evaluated based on high-resolution CT (HRCT) in a series of 103 patients with MD (93 unilateral cases and 10 bilateral cases). JB-related indices included JB anteroposterior and mediolateral diameter, JB height, JB type regarding to Manjila classification system, and incidences of JB diverticulum (JBD), JB related inner ear dehiscence (JBID), and inner ear adjacent JB (IAJB). VA-related indices included CT-VA visibility, CT-VA morphology (funnel, tubular, filiform, hollow, and obliterated-shaped type), and peri-VA pneumatization. Radiological indices were compared between MD ears and control ears.

**Results:**

Radiological JB abnormalities were comparable between MD ears and control ears. As for VA-related indices, the CT-VA visibility was lower in MD ears than in control ears (*p* = 0.004). The distribution of CT-VA morphology was significantly different between MD and control ears (*p* = 0.013), with a higher proportion of obliterated-shaped type in MD ears (22.1%) than in control ears (6.6%).

**Conclusion:**

Compared with JB abnormalities, the anatomical variations of VA are more likely to be an anatomically predisposing factor for MD.

## 1. Introduction

Ménière disease (MD) is a complex inner ear disease histopathologically characterized by idiopathic endolymphatic hydrops (ELH), with episodes vertigo attacks, fluctuating sensorineural hearing loss, tinnitus, and aural fullness as the main clinical symptoms. The etiology of the disease has not been fully elucidated. It is now believed that MD may be related to excessive endolymph production and/or compromised endolymph absorption by the endolymphatic duct (ED) and endolymphatic sac (ES). The underlying pathophysiological mechanisms may involve anatomical variation, autoimmunity, viral infection, genetics, ionic imbalance, vascular irregularities, allergic responses, and others (1). Among them, the anatomical variations of the inner ear have been extensively studied. Histopathologically, Ikeda and Sando found that the vestibular aqueduct (VA) and ES were shorter in MD patients compared to healthy individuals (2). Similar findings have been highlighted by radiological investigations using computed tomography (CT) and magnetic resonance imaging (MRI), which described a link between invisible VA or ED and the clinical course of MD (3, 4).

Besides the VA, previous studies have suggested a correlation between MD and anatomical anomalies in jugular bulb (JB) (5). JB abnormalities usually include the high JB (HJB), JB diverticulum (JBD), inner ear adjacent JB (IAJB), JB adjacent to inner ear dehiscence (JBID). Park et al. found that MD patients had a higher incidence of HJB, JBD and IAJB compared to normal individuals (6). Several studies have suggested that HJB may lead to ELH by compressing VA or ES, causing MD-like symptoms (6–9). Furthermore, Couloigner et al. (10) surgically reduced the height of the JB in MD patients with HJB, which significantly relieved vertigo symptoms, thus confirming the potential relationship between HJB and MD. It has been proposed that JB abnormalities may impair endolymph absorption through direct (compression of the ED or ES) or indirect (compression of the venous drainage of the ED and/or ES) effects (10), thus leading to ELH. However, this hypothesis has been questioned by other studies. In a series of MD patients confirmed by gadolinium-enhanced MRI (Gd-MRI) of the inner ear, Oya et al. (11) examined the incidence of JB abnormalities between the affected side of MD, the non-affected side of MD, and the control group, and found no differences in the incidence of HJB and JB surface area among the three groups.

To our knowledge, few studies have investigated the relevance of JB abnormalities and VA variations in the same MD cohort (8, 9). Karatas et al. (8) had measured JB anomalies and ED length and width in patients with unilateral MD. However, only a small sample was enrolled and few imaging variables were analyzed, and the incidences of JB and VA variations were not compared (8).

In this retrospective study, anatomical variations of JB and VA were evaluated based on high-resolution CT (HRCT) in patients with clinically diagnosed MD. We sought to gain deeper insight into the clinical significance of anatomical factors in MD.

## 2. Materials and methods

### 2.1. Study population

This retrospective chart review was conducted in Union Hospital affiliated to Tongji Medical College, Huazhong University of Science and Technology, Wuhan, China.

One hundred and three patients with definite MD (93 unilateral MD patients, 10 bilateral MD patients) were enrolled between September 2013 and December 2020. All patients underwent a thorough history inquiry, otoscopy, neurotological evaluations (audiometry, impedance, videonystagmograph, caloric test, etc.) and imaging examination for differential diagnosis. The diagnosis of MD was established following the diagnostic guidelines of MD outlined by the American Academy of Otolaryngology-Head and Neck Surgery (AAO-HNS) in 1995. Those MD patients were considered to be ineligible to participate in the study if they had: (1) middle or inner ear malformations; (2) middle or inner ear infections (otitis media, mastoiditis, labyrinthitis etc.); (3) retro-cochlear lesions (vestibular schwannoma, internal acoustic canal stenosis etc.); (4) history of ear surgery or intratympanic injections; (5) systemic diseases; (6) disorders of central nervous system (vestibular migraine, multiple sclerosis, cerebellar infarction, etc.). The control group included patients with suspected head trauma. Those ears with temporal bone fracture, previous otitis media, and hearing loss were excluded, resulting in a total of 106 control ears.

This study was conducted in compliance with the tenets of the Declaration of Helsinki. Informed consent was obtained from each patient and control. The project was approved by the ethical committee of Union Hospital, Tongji Medical College, Huazhong University of Science and Technology.

### 2.2. Radiological evaluations

#### 2.2.1. High-resolution CT protocol

All patients were scanned while supine in a craniocaudal direction using a 64-detector spiral CT scanner (Somatom Defnition AS+, Siemens, Germany). The scan plane was parallel to the orbitomeatal line. Scan parameters were as follows: tube voltage 120 kV, CASE Dose 4D quality reference mAs: 180 mAs, slice thickness 0.6 mm, slice collimation 128 × 0.6 mm, pitch 0.5, field of view 150 mm, reconstruction increment 0.3 mm and reconstruction kernel H60s.

All CT images were transferred and analyzed on a picture archiving and communication system (PACS) workstation (Carestream Client, Carestream Health). Multi-plane reconstruction in axial, coronal, and sagittal planes were performed. Radiological data were reviewed by two senior neuroradiologist who were blinded to the clinical data (PL with an experience of over 10 years and JL of over 5 years).

#### 2.2.2. Radiological measurement

**Figure 1:**
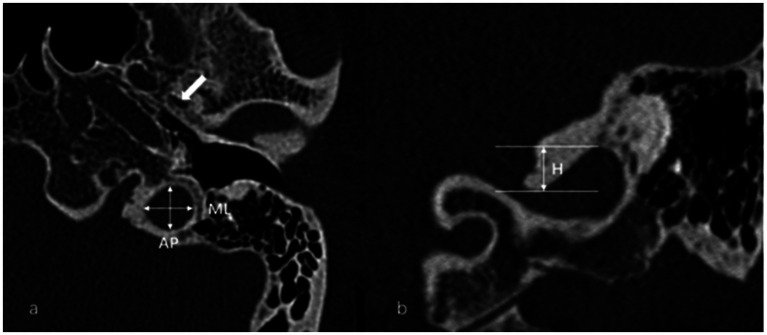
**(a)** Anteroposterior (AP) and mediolateral (ML) diameters of the jugular bulb (JB) were measured on the axial image at the level where the foramen spinosum (arrow) could be observed. **(b)** The height (H) of the JB was determined by measuring the distance between the level of the JB dome and the line passing through the confluence of the sigmoid sinus with the JB on the coronal image.

**Figure 2:**
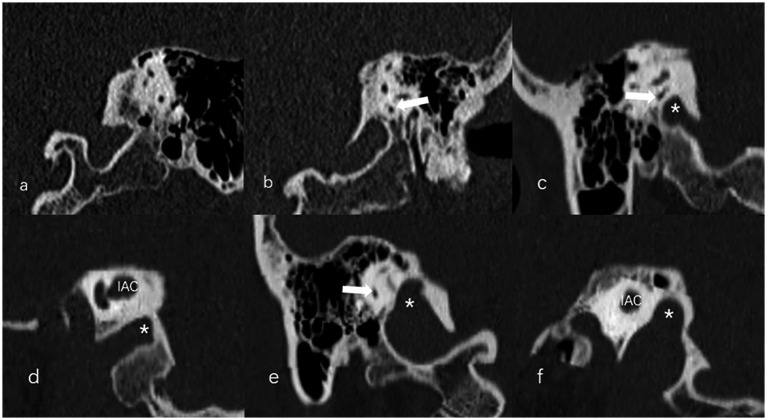
The Manjila classification of jugular bulb (JB) (asterisk) location. **(a)** Type 1, no bulb; **(b)** Type 2, below the inferior margin of the posterior semicircular canal (PSCC) (arrow); **(c,d)** Type 3, between the inferior margin of the PSCC (arrow) and the inferior margin of the internal auditory canal (IAC); **(e,f)** Type 4, above the inferior margin of the IAC.

**Figure 3:**
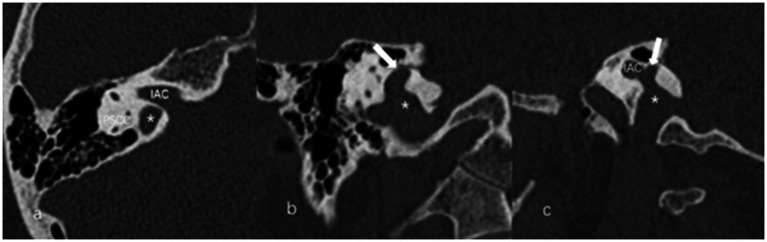
The axial **(a)**, coronal **(b),** and sagittal **(c)** images of jugular bulb diverticulum (JBD). **(a)** High jugular bulb (asterisk) lies in the triangular area between the internal auditory canal (IAC), posterior semicircular canal (PSCC) and posterior surface of the petrous bone. **(b,c)** Jugular bulb diverticulum is displayed as a vertical outpouching (arrow) from the jugular bulb (asterisk) on the coronal and sagittal image of temporal bone high-resolution computed tomography.

**Figure 4:**
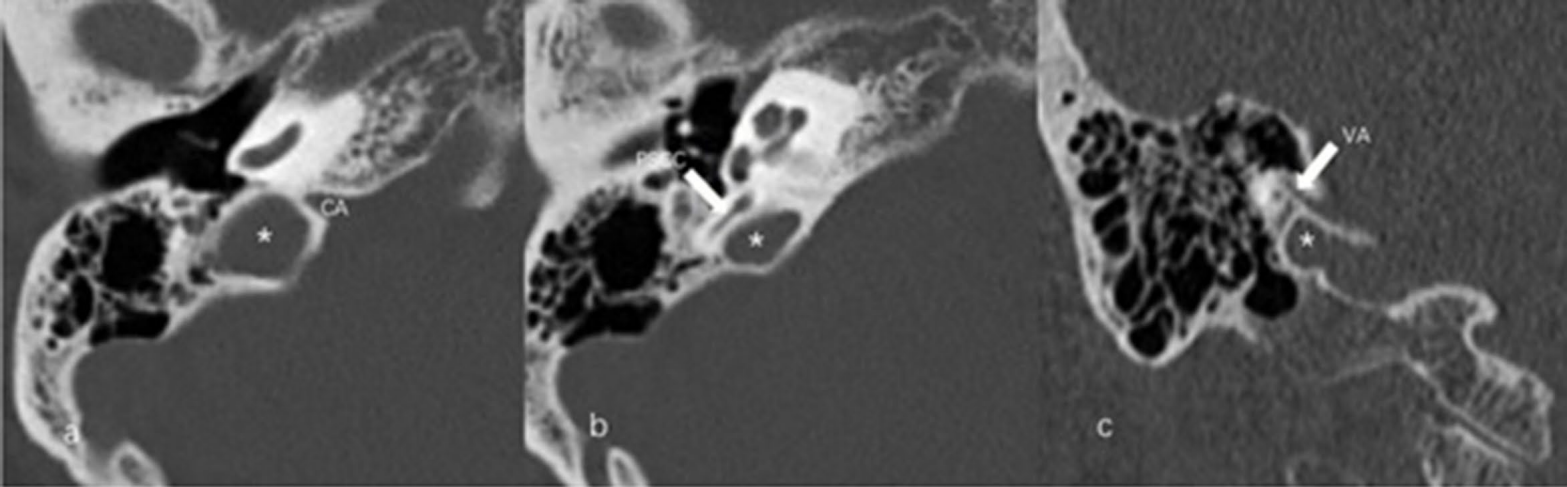
Inner ear adjacent jugular bulb (IAJB). **(a)** Jugular bulb (JB) (asterisk) is adjacent to the cochlear aqueduct (CA) on the axial image of temporal bone high-resolution computed tomography (HRCT). **(b)** JB (asterisk) is adjacent to the posterior semicircular canal (arrow) on the axial image of temporal bone HRCT. **(c)** JB (asterisk) is adjacent to the vestibular aqueduct (arrow) on the coronal image of temporal bone HRCT.

**Figure 5:**
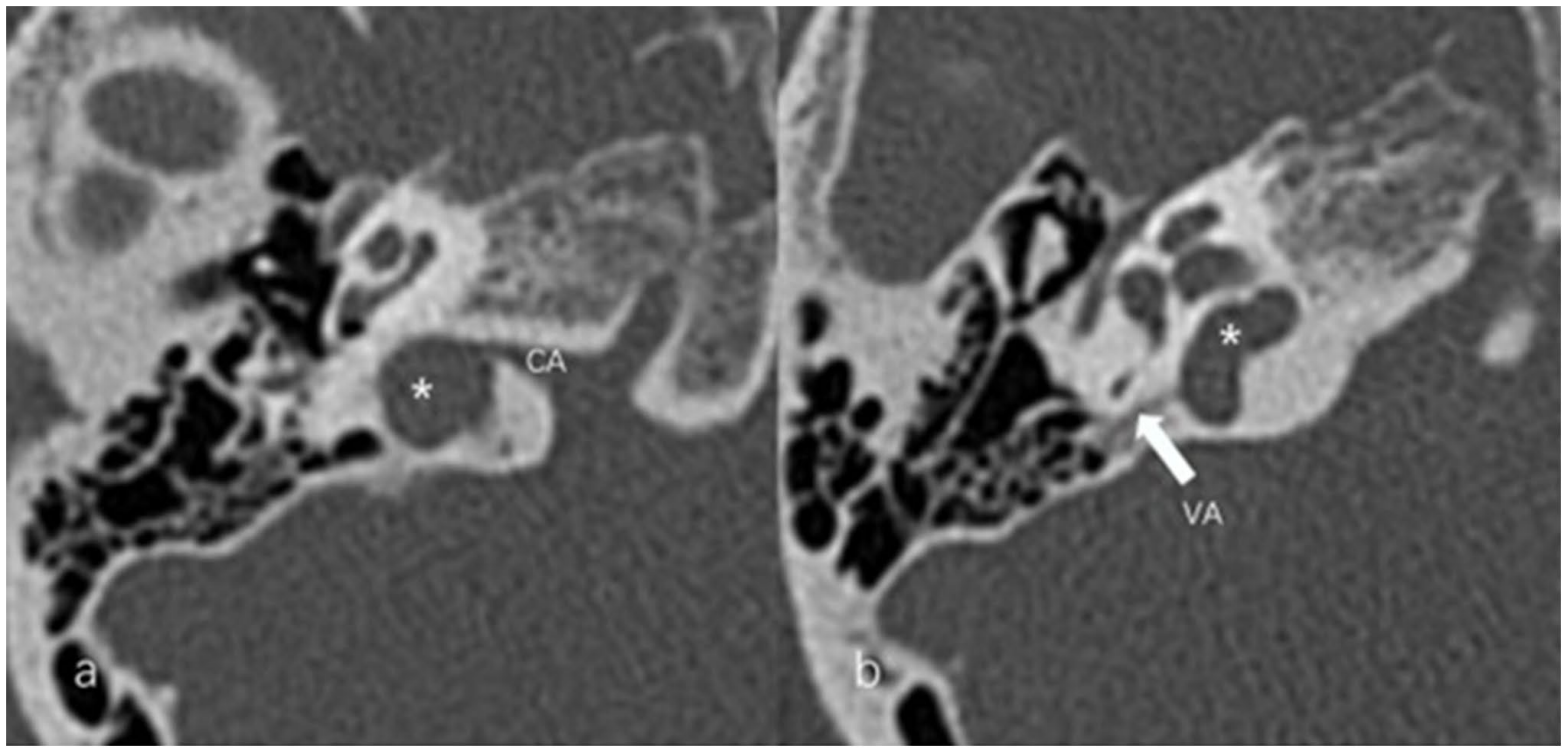
Jugular bulb related inner ear dehiscence (JBID). **(a)** Bony dehiscence between jugular bulb (JB) (asterisk) and the cochlear aqueduct (CA) on the axial image of temporal bone high-resolution computed tomography (HRCT). **(b)** Bony dehiscence between JB (asterisk) and the vestibular aqueduct (arrow) on the axial image of temporal bone HRCT.

The anteroposterior (AP) and mediolateral (ML) diameters of JB were measured on the axial image parallel to the orbitomeatal line at the level where the foramen spinosum could be observed (Figure 1a). The distance between the JB dome and the confluence of the sigmoid sinus on the coronal image was defined as the height of JB (Figure 1b). We used the Manjila classification system to describe the anatomical location of JB (12) (Figure 2): type 1, no bulb; type 2, below the inferior margin of the posterior semicircular canal (PSCC); type 3, between the inferior margin of the PSCC and the inferior margin of the internal auditory canal (IAC); type 4, above the inferior margin of the IAC. Other types of JB abnormalities included: (1) jugular bulb diverticulum (JBD): a prominent protrusion or outpouching of JB forming a smooth ellipsoidal form (Figure 3); (2) inner ear adjacent jugular bulb (IAJB): an ossification between JB and VA, CA, PSCC less than 1 mm (Figure 4). IAJB was subdivided into cochlear aqueduct adjacent jugular bulb (CAAJB) (Figure 4a), PSCC adjacent jugular bulb (PSAJB) (Figure 4b), and vestibular aqueduct adjacent jugular bulb (VAAJB) (Figure 4c), respectively; (3) jugular bulb related inner ear dehiscence (JBID): a deossification between JB and VA, CA, PSCC (Figure 5). JBID was subdivided into jugular bulb related vestibular aqueduct dehiscence (JBVAD), jugular bulb related cochlear aqueduct dehiscence (JBCAD), jugular bulb related PSCC dehiscence (JBPSD).

**Figure 6:**
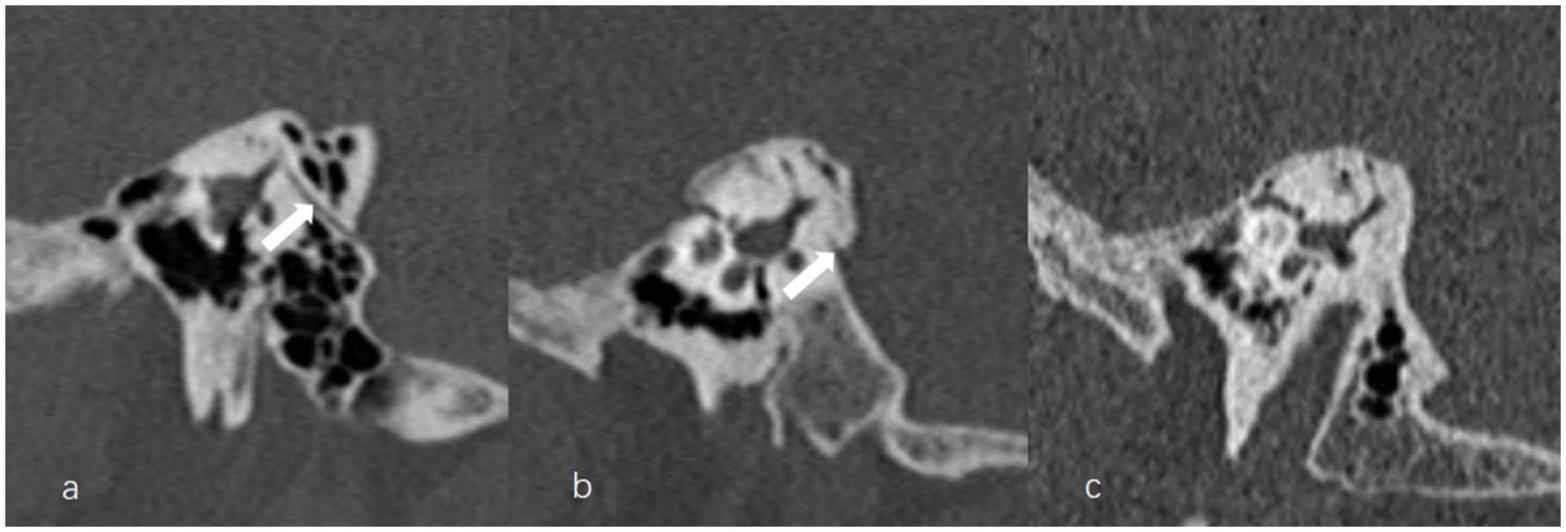
The grading of vestibular aqueduct (VA) visibility on the 45° oblique (Pöschl) planes on temporal bone computed tomography. **(a)** Grade 0 with a continuous VA (arrow). **(b)** Grade I with a discontinuous VA (arrow). **(c)** Grade II with a complete ossification of VA.

**Figure 7:**
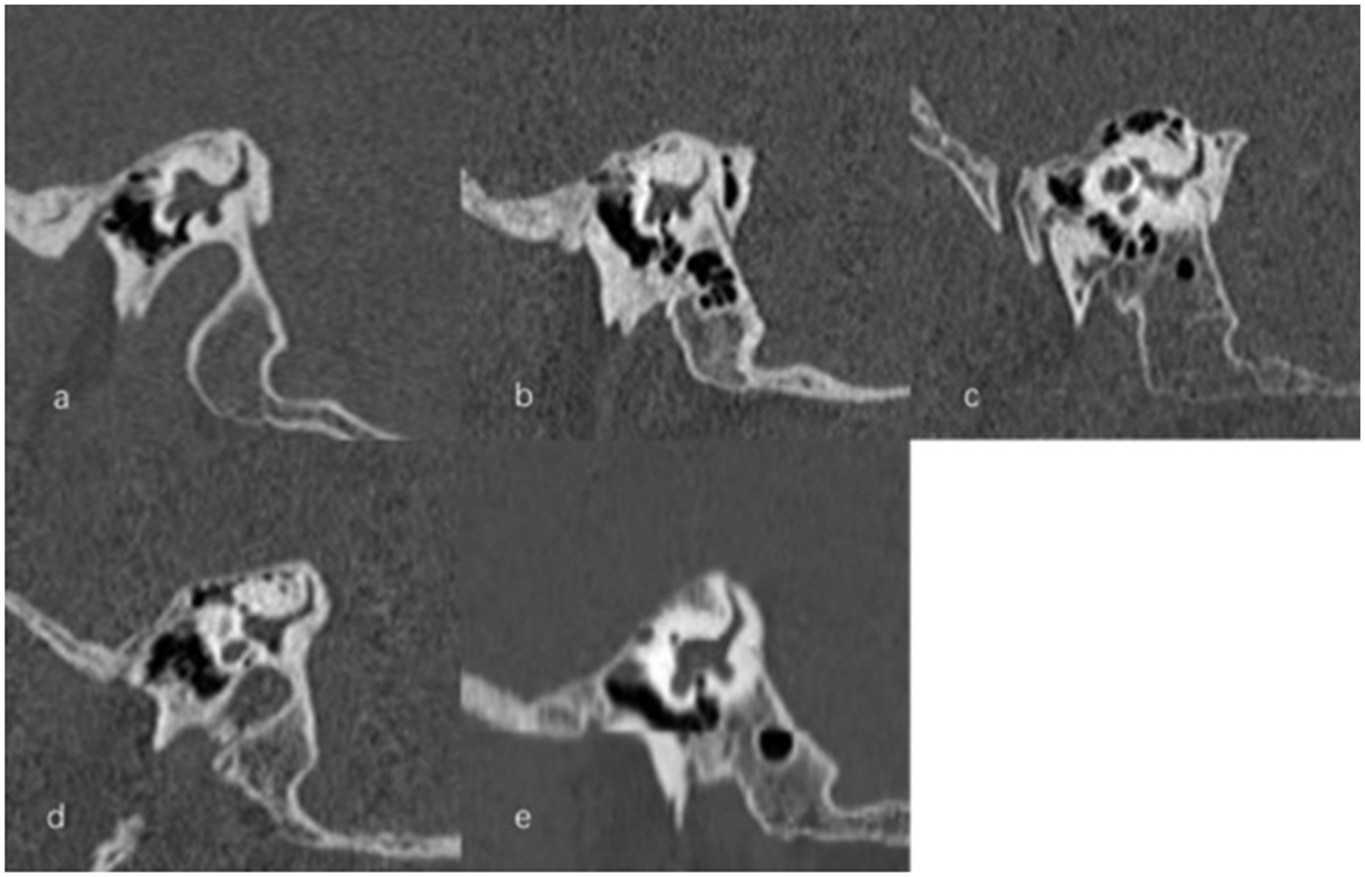
Types of vestibular aqueduct (VA) morphology. **(a)** Funnel-shaped type; **(b)** Tubular-shaped type; **(c)** Filiform-shaped type; **(d)** Hollow-shaped type; **(e)** Obliterated-shaped type.

**Figure 8:**
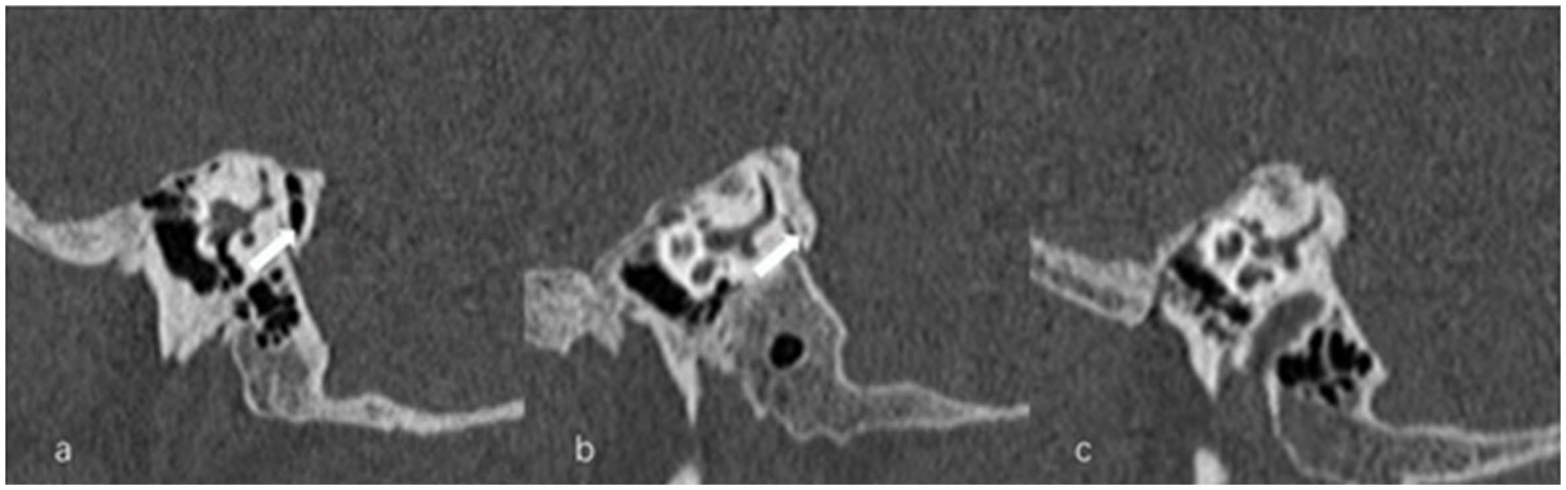
Types of peri-vestibular aqueduct (VA) pneumatization. **(a)** Large-cell pneumatization (arrow) in the vicinity of the VA, **(b)** small-cell pneumatization (arrow) in the vicinity of the VA, **(c)** absence of air cells.

The visibility of VA in HRCT was graded as previously reported (13): grade 0, continuous VA; grade I, discontinuous VA; grade II, invisible VA (Figure 6). We used Yamane classification criteria to describe the morphology of VA (4): (A) funnel type, (B) tubular type, (C) filiform type, (D) hollow type, (E) obliterated type (Figure 7). Peri-VA pneumatization were categorized as type 1: large-cell pneumatization in the vicinity of the VA; type 2: small-cell pneumatization in the vicinity of the VA; and type 3: absence of air cells (Figure 8) (14).

### 2.3. Statistical analysis

Data were analyzed and processed by SPSS 25.0 statistical software. Continuous variables were expressed as mean ± standard deviation or median (quartile). Categorical variables and grade variables were expressed as frequency (percentage). The normality of data was assessed by Shapiro – Wilk test. A *t*-test was used to compare continuous variables of two independent samples with normal distribution. A Mann–Whitney *U* test was used to compare grade variables or continuous variables of skewed distribution between two independent samples. Categorical variables were compared using Chi square test or Fisher’s exact test for two independent samples. The inter-rater reliability of the radiological results was determined using intra group correlation coefficient (ICC) for measurement data and kappa value for count data and grade data, respectively. The interpretation of agreement was as follows: ICC ≤ 0.20, poor; 0.2 < ICC ≤ 0.40, fair; 0.4 < ICC ≤ 0.60, moderate; 0.6 < ICC ≤ 0.80, good; 0.8 < ICC ≤ 1.0, excellent. Kappa ≤0.20, poor; 0.21 < kappa ≤0.40, fair; 0.41 < kappa ≤0.60, moderate; 0.61 < kappa ≤0.80, good; 0.81 < kappa ≤1.0, excellent. Good to excellent inter-observer agreement was found in our radiological evaluation regarding to JB and VA variations. Statistical significance was set as *p* < 0.05.

## 3. Results

### 3.1. Demographic characteristics of the participants

In this study, 176 subjects were enrolled, including 103 MD patients (93 unilateral cases and 10 bilateral cases) and 73 control subjects. The median age of MD patients was 53 (47, 59) years. The male/female ratio was 38/65, and the median course of disease was 2 (0.5, 3) years. The average age of the control group was 47.2 ± 16.1 years. The male/female ratio was 32/41.

### 3.2. Size of JB in MD patients

According to Manjila classification system (12), for all 113 MD ears involved, type 1 JB was identified in 34 ears (30.1%), type 2 in 56 ears (49.6%), type 3 in 16 ears (14.2%), and type 4 in 7 ears (6.2%). For all 106 control ears, type 1 JB was found in 27 ears (25.5%), type 2 in 61 ears (57.5%), type 3 in 9 ears (8.5%) and type 4 in 9 ears (8.5%). There was no significant difference in the type of JB between MD ears and control ears (Z = −0.281, *p* = 0.779).

For all participants involved, the AP and ML diameters could not be obtained in 49 ears with Manjila type 1 JB and 19 ears with type 2 JB, including 33 MD ears and 35 control ears, for the dome of JB did not reach the level of the foramen spinosum (9). The height of Manjila type 1 JB was unavailable in 34 MD ears and 27 control ears. Therefore, the radiological data of JB size from 80 MD ears and 71 control ears were measured and analyzed. The AP diameter of JB was 7.895 ± 2.187 mm on the MD ears and 7.879 ± 2.178 mm on the control ears. The ML diameter of JB was 9.368 ± 3.095 mm on the MD ears and 8.755 ± 2.438 mm on the control ears. Also, radiological data of JB height from 79 MD ears and 79 control ears were obtained for analysis and it was 4.498 ± 2.142 mm on the MD ears and 4.340 (2.600, 5.820) mm on the control ears. None of these JB-related variables differed between the MD ears and control ears (*t* = 0.045, *p* = 0.964 for AP diameter; *t* = 1.360, *p* = 0.176 for ML diameter; Z = −0.539, *p* = 0.590 for height).

### 3.3. Jugular bulb abnormalities in MD patients

The prevalence of JBD, JBID and IAJB were 9.7% (11/113), 6.2% (7/113) and 23% (26/113) in all MD ears, whereas it was 4.7% (5/106), 7.5% (8/106) and 17% (18/106) in control ears, respectively. We observed co-existence of multiple subtypes of JBID in 3 MD ears and 1 control ear. The incidences of JBVAD, JBCAD, and JBPSD were 3.5% (4/113), 4.4% (5/113), and 0.9% (1/113) in all MD ears involved, whereas it was 6.6% (7/106), 1.9% (2/106) and 0 in control ears, respectively. For the subtypes of IAJB, multiple subtypes of IAJB coexisted in 8 MD ears and 7 control ears. The incidences of VAAJB, CAAJB and PSAJB were 3.5% (4/113), 13.3% (15/113), and 13.3% (15/113) in MD ears, whereas it was 3.8% (4/106), 11.3% (12/106), and 9.4% (10/106) in control ears, respectively. No significant differences were found in the above comparisons (Table 1).

**Table 1:** Radiological incidences of jugular bulb abnormalities in MD patients and control subjects.

	MD ears (*n* = 113)	Control ears (*n* = 106)	*x* ^2^	*p*
JBD	11 (9.7%)	5 (4.7%)	2.033	0.154
JBID	7 (6.2%)	8 (7.5%)	0.157	0.692
JBVAD	4 (3.5%)	7 (6.6%)	1.076	0.300
JBCAD	5 (4.4%)	2 (1.9%)	0.466	0.495
JBPSD	1 (0.9%)	0 (0.0%)	NA	1.0
IAJB	26 (23%)	18 (17%)	1.238	0.266
VAAJB	4 (3.5%)	4 (3.8%)	NA	1.0
CAAJB	15 (13.3%)	12 (11.3%)	0.193	0.660
PSAJB	15 (13.3%)	10 (9.4%)	0.798	0.372

JBD, jugular bulb diverticulum; JBID, jugular bulb related inner ear dehiscence; JBVAD, jugular bulb related vestibular aqueduct dehiscence; JBCAD, jugular bulb related cochlear aqueduct dehiscence; JBPSD, jugular bulb related posterior semicircular canal dehiscence; IAJB, inner ear adjacent jugular bulb; VAAJB, vestibular aqueduct adjacent jugular bulb; CAAJB, cochlear aqueduct adjacent jugular bulb; PSAJB, posterior semicircular canal adjacent jugular bulb; NA, not applicable.

### 3.4. Vestibular aqueduct-related variations in MD patients

As described in Table 2, CT-VA visibility was rated as grade 0 and grade I in 73.5% (83/113) and 26.5% (30/113) of all MD ears, respectively, and in 88.7% (94/106) and 11.3% (12/106) of the control ears, respectively. Grade II (CT-VA invisibility) was not observed in either MD patients or control subjects. VA visualization was poorer in the MD ears than in the control ears (Z = −2.854, *p* = 0.004).

**Table 2:** Radiological incidences of variables of VA in MD patients and control subjects.

	MD ears (*n* = 113)	Control ears (*n* = 106)	Z/*x*^2^	*p*
Grade 0	83 (73.5%)	94 (88.7%)	Z = –2.854	0.004
Grade I	30 (26.5%)	12 (11.3%)		
Grade II	0 (0.0%)	0 (0.0%)		
Type A: funnel	20 (17.7%)	18 (17.0%)	$x^{2}$=11.802	0.013
Type B: tubular	34 (30.1%)	43 (40.6%)		
Type C: filiform	33 (29.2%)	37 (34.9%)		
Type D: hollow	1 (0.9%)	1 (0.9%)		
Type E: obliterated	25 (22.1%)	7 (6.6%)		
Type 1	20 (17.7%)	32 (30.2%)	Z = –0.560	0.576
Type 2	29 (25.7%)	12 (11.3%)		
Type 3	64 (56.6%)	62 (58.5%)		

According to the Yamane classification criteria, type A, B, C, D, and E were found in 17.7% (20/113), 30.1% (34/113), 29.2% (33/113), 0.9% (1/113), and 22.1% (25/113) of the MD ears, respectively, whereas the corresponding incidence was 17.0% (18/106), 40.6% (43/106), 34.9% (37/106), 0.9% (1/106), and 6.6% (7/106) in the control ears, respectively. Compared with the control ears, the shape of VA differed considerably between the MD ears and the control ears ($x^{2}\;$= 11.802, *p* = 0.013). Type E (obliterated) was more prevalent in MD ear ($x^{2}\;$= 10.559, *p* = 0.001) (in Table 2).

For radiological peri-VA pneumatization, type 1 (large-cell), 2 (small-cell) and 3 (absence of air cells) were demonstrated in 17.7% (20/113), 25.7% (29/113), and 56.6% (64/113) of the MD ears, and in 30.2% (32/106), 11.3% (12/106) and 58.5% (62/106) of the control ears. The MD ears and the control ears showed similar pattern of peri-VA pneumatization (Z = −0.560, *p* = 0.576) (in Table 2).

## 4. Discussion

### 4.1. Jugular bulb abnormalities in MD patients

The JB is an enlarged confluence connecting the sigmoid sinus and the internal jugular vein, lying in the triangular area between the inner acoustic meatus, the PSCC, and the posterior surface of the petrous bone. ELH, the histopathological hallmark of MD, can be caused by excessive secretion of endolymph and/or deficient reabsorption of endolymph through the ED and ES. JB abnormalities might cause hearing loss, vertigo, and pulsatile tinnitus. Moreover, they can cause MD and ELH. It has been reported that the contact between high JB with VA was more prevalent in MD patients than in control subjects (10). However, other studies demonstrated no association between the JB abnormalities and audio-vestibular symptoms or MD (11, 15). In this study, all radiological indices associated with the JB abnormalities were comparable between MD ears and control ears. Thus, the role of JB abnormalities in MD remained controversial (11).

#### 4.1.1. Size of JB in MD

Our results found that both the MD ears and the control ears had comparable JB measures in terms of height and types based on the Manjila classification system. Anatomical variation of JB location, especially the HJB, has been explored in the pathogenesis of MD. Recently, Park et al. showed that the frequencies of HJB were higher in MD group compared to control group (6). However, in a cohort of MD patients with MRI-demonstratable ELH, Oya et al. found that the detection rate of HJB did not differ between the MD affected side, MD non-affected side, and control subjects (11). The disagreement in the incidence of HJB may be attributed to large variation in the definition of HJB (16). To date, HJB has been variably defined as a JB which reaches the lower margin of round window, the basal turn of the cochlea, the floor of hypotympanum, the IAC, or 2 mm below the IAC, etc. (17–20). Given the lack of consensus on the definition of HJB, Manjila et al. proposed a novel grading system in terms of the anatomical location of the JB (12). Using this new grading system, Hu et al. (9) showed similar incidence of types 1, 2, and 3 JB between ELH and non-ELH ears confirmed by the Gd-MRI of inner ear in MD patients, while the presence of type 4 JB and height of the JB differed between these two sub-groups.

By surgically depressing the JB, Couloigner et al. showed that vertigo episodes disappeared in 54% (7/13) and vertigo intensity decreased in 38% (5/13) of the patients with high and medial JB associated with MD and pulsatile tinnitus (10). Recently, in three patients presenting a HJB or a JBD with dehiscence and compression of the VA, Hitier et al. showed that the disabling vertigo induced by JB abnormalities can be treated effectively by using an endovascular technique to plug the upper part of JB (21). Therefore, pathophysiological relevance of HJB in MD and other vestibular disorders warrants further investigations, and future studies are needed to validate the clinical value of the Manjila classification system.

In our series, no difference in AP or ML diameters of the JB was found between MD ears and control ears, which were consistent with the results of Hu et al. (9). Moreover, Oya et al. detected no difference in JB surface area between MD ears and healthy ears (11), which was derived from AP and ML measurement (22).

#### 4.1.2. Other JB abnormalities in MD

Our results revealed no differences in incidences of JBD, JBID, and IAJB between the MD ears and the control ears. To date, few studies have investigated the associations between MD and JBD, JBID, and IAJB. With HRCT, Park et al. found that the frequencies of JBD and IAJB were higher in MD group compared to control group (6), while no differences in JBID were observed between both groups. The disagreement between our results and those of Park et al. may be attributed to the different inclusion criteria.

In this study, JBID was further classified into three subtypes, i.e., JBVAD, JBCAD, and JBPSD, and the highest incidence of JBVAD was found in the control ear (87.5%, 7/8). Friedmann et al. found 44 JBID in 1579 temporal bone specimens, and 93.2% (41/44) JBIDs was classified as JBVAD (23). In a cohort of patients with inner ear symptoms or facial palsy, Park et al. identified 21 JBIDs in 552 ears, in which JBVAD was the most frequent, constituting 90.1% (19/21) of all JBIDs (24). In our study, the prevalence of JBVAD in control ears is parallel with the above findings. The comparable prevalence of JBVAD subtype and JBCAD subtype in MD ears may be caused by a relatively small sample size in our series.

### 4.2. Radiological variations of VA in MD patients

#### 4.2.1. CT-VA visibility

In this study, MD ears had a lower rate of the CT-VA visibility compared with control ears, which is consistent with the previous studies. Atrophy of the ES, hypoplasia of the VA and narrowing of the lumen of the ED has been observed in the temporal bone specimens of MD patients. It is believed that hypoplasia of ES and ED impairs endolymph absorption, which may precipitate ELH in MD. Therefore, congenital or developmental abnormality of the VA/ED has been considered as a likely predisposing factor for the development of ELH in patients with MD. Similar findings were highlighted radiologically, as demonstrated by the lack of visible VA or ED (3, 13). To date, several radiological techniques have been used to evaluate and grade the visibility of VA in MD patients, including CT, CBCT and MRI (4, 25, 26). Numerous studies have consistently reported significantly reduced radiological VA visibility in the affected ear with MD compared to the healthy ear.

Recently, CT and Gd-MRI of the inner ear was used in combination to assess the visualization of the VA and ELH *in vivo* simultaneously in MD patients. Using this radiological procedure, Mainnemarre et al. identified invisibility of VA as a predictor for the presence of saccular hydrops with a positive predictive value of 93.1% (13). Similarly, Grosser et al. detected a significant relationship between the invisibility of CT-VA and the degree of the cochlear hydrops *in vivo* (25). Therefore, VA may be critically involved in the pathophysiological process of MD, which not only promote the initiation but also the progression of ELH in patient with MD.

#### 4.2.2. Vestibular aqueduct morphology

In addition to VA visibility, the morphology of VA has been evaluated radiologically in terms of length, width, area, and shape (4, 27). In our series, MD ears had a higher proportion of obliterated VA than control ears. Traditionally, CT-VA morphology is classified as funnel, tubular, and filiform-shaped (28). Yamane et al. refined the classification criteria by adding two novel types, i.e., hollow and obliterated types, and found that the obliterated type of VA is characteristic in the MD affected ears (4). Our results are consistent with the findings of Yamane et al. (4), indicating that a more sophisticated imaging evaluation could better estimate the VA function in patients with MD.

VA morphology has recently been demonstrated to correlate with ES pathologies. In the pilot study by Bächinger et al., the angular trajectory of the VA (ATVA) on CT could be used as a surrogate imaging marker of ES pathologies in MD, namely, degeneration and hypoplasia (29), which are directly linked to different ELH pathogenesis and MD clinical features. For example, MD subgroup with degenerative ES presented a higher level of vertigo attacks and poorer vestibular function in terms of caloric asymmetry, while the MD subgroup with hypoplastic ES presented a male preponderance, higher frequencies of bilateral involvement, a positive family history for hearing loss/vertigo/MD (30, 31). ES is essential in maintaining endolymphatic homeostasis by reabsorption and secretion (32, 33), and it is also the primary site of immune defense in the inner ear (34). Therefore, ES may play multiple roles in the pathogenesis in ELH and ES pathologies may contribute to the heterogeneity of clinical features and therapeutic response in MD. Recently, a novel surgical technique, endolymphatic duct blockage (EDB), has been devised for treating refractory MD with the rationale that blocking ED could eliminate endolymph backflow caused by ES hypersecretion (35). Wang et al. found that the therapeutic efficacy of EDB was correlated with the pathology of extraosseous ES (eES), that is, compared with MD patients with atrophic eES, those with normoplastic eES were more likely to have complete vertigo control, better audio-vestibular function and milder vestibular ELH postoperatively. The authors attributed the difference in response to EDB surgery to the functional status of ES and the pathogenesis of ELH, i.e., a normoplastic eES may indicate endolymph hypersecretion as the predominant cause of ELH whereas an atrophic eES may indicate that endolymph malabsorption play a major role (36). Therefore, elaborate assessment of the VA morphology may provide a deeper insight into the pathophysiological mechanisms of MD, which could in turn promote pathogenesis-oriented treatment.

We have noticed that a small proportion of normal controls also exhibited obliterated type of VA. This finding was not totally unexpected. Some investigations have suggested that the presence of ELH in MD might be an epiphenomenon, as patients with ELH do not necessarily present the classical symptoms of MD (1, 37). Animal studies have shown that only ELH is insufficient to cause typical acute balance disturbance and spontaneous nystagmus observed in MD (38, 39). Combined actions of other etiological factors are mandatory, such as reduction of inner ear blood flow. Therefore, we speculate that obliterated VA on CT examination is insufficient to trigger MD in normal controls as other stress factors may be missing. Of note, narrower VA and/or ED indicates a compromised functional reserve of endolymphatic homeostasis (40) and those normal controls with an obliterated type VA may be more susceptible to develop MD. Due to the cross-sectional nature of this study, the prognosis of the normal controls with obliterated type VA requires further prospective investigation.

#### 4.2.3. Peri-VA pneumatization

We found similar pattern of peri-VA pneumatization between MD ears and control ears. Previous studies have yielded conflicting results on this issue. With tomogram, Stahle and Wilbrand (41) demonstrated poor peri-VA pneumatization in MD patients compared to control subjects, and an association between the peri-VA pneumatization and VA length. They also detected a reduction in size of the mastoid air cell system in MD patients using the tomography and plain radiography (14). In contrast, in a cohort of MD patients confirmed by Gd-MRI of inner ear, Oya et al. found no difference in pneumatization of peri-VA air cells, measured by CT, among the MD affected ears, the non-affected ears, and the controls (11). The discrepancies in these findings may be attributed to differences in inclusion criteria and radiological techniques.

### 4.3. Strength and limitation

Several radiological studies have examined the presence of anatomical variations of inner ear in patients with MD. These variations included shorter distance between the vertical part of the PSCC and the posterior fossa (26), less visibility of VA or ED (42), higher prevalence of JB abnormalities (6), poorer peri-VA pneumatization (43), retro-vestibular bony hypoplasia (44), and so on. To the best of our knowledge, this study is the first to simultaneously investigate the anatomical variations of JB and VA in the same MD cohort. Our results demonstrated that, compared with JB abnormality, the VA-related variation is more consistent and prominent in MD patients, which may provide a basis for radiomic feature identification and quantitative analysis in future radiological study.

It is important to mention that, JB abnormalities are currently believed to impact MD through their effect on VA. Couloigner et al. postulated that JB abnormalities could induce MD by a direct or indirect effect on the ED and/or ES, producing a decrease in endolymph resorption (10). This decrease of endolymph resorption may be due to compression of the ED or ES (direct effect) or compression of the venous drainage of the ED and/or ES (indirect effect). Indeed, recent histopathological and imaging studies have discovered a rich lympho-venous plexus surrounding ED, which drains distally into the vein of VA and finally into the sigmoid sinus or JB. This peri-ED plexus is believed to be crucial for endolymphatic resorption (45, 46). In this sense, JB variation is not an independent anatomical factor as it might induce MD through its link with VA. In this study, the incidences of JB-VA relationship indices, such as JBVAD and VAAJB, did not differ between MD ears and control ears, indicating that JB abnormalities and its related VA changes may be infrequent in MD patients.

Several limitations were present in this study. Firstly, vestibular tests such as vestibular evoked myogenic potentials (VEMPs) and evoked nystagmus were not performed in our series. As Hitier et al. suggested, clinically, it is important to differentiate between those JB abnormalities that are responsible for MD and those which are fortuitous associations (47). Four clues have been proposed to assess this potential association: (1) vertigo attacks induced by high venous pressure (coughing or Valsalva maneuvers) or intense sound (Tullio phenomenon); (2) pulsatile tinnitus; (3) an induced nystagmus during the Valsalva maneuver; and (4) reduced VEMPs threshold resembling the third window phenomenon (48, 49). Secondly, the diagnosis of MD in this study was based on clinical manifestations and pure tone audiometry, and ELH was not confirmed by the Gd-MRI of inner ear. Thirdly, only a few bilateral cases were enrolled in this study. Therefore, future study including full spectrum of MD subtypes and comprehensive vestibular tests are warranted to further understand the impact of anatomical variations on MD.

## 5. Conclusion

The anatomical variations of VA are more likely to be an anatomically predisposing factor for MD than abnormalities of JB.

## Data availability statement

The original contributions presented in the study are included in the article/Supplementary material, further inquiries can be directed to the corresponding authors.

## Ethics statement

The studies involving human participants were reviewed and approved by the ethical committee of Union Hospital, Tongji Medical College, Huazhong University of Science and Technology. The patients/participants provided their written informed consent to participate in this study.

## Author contributions

KX: interpretation of data, statistical analysis, drafting and critical revision of the manuscript. PL: interpretation of data, image extraction and analysis, and critical revision of the manuscript. YZL: patient recruitment and statistical analysis. JL: data collection and image extraction and analysis. MW and YML: patient recruitment, patient consultation, and data collection. BL: study conception and design, patient consultation, interpretation of data, and critical revision of the manuscript. All authors read and approved the final manuscript.

## Funding

This work was supported by grants from the National Natural Science Foundation of China (No. 81670930) and the Natural Science Foundation of Hubei Province, China (No. 2021CFB547).

## Conflict of interest

The authors declare that the research was conducted in the absence of any commercial or financial relationships that could be construed as a potential conflict of interest.

## Publisher’s note

All claims expressed in this article are solely those of the authors and do not necessarily represent those of their affiliated organizations, or those of the publisher, the editors and the reviewers. Any product that may be evaluated in this article, or claim that may be made by its manufacturer, is not guaranteed or endorsed by the publisher.
